# Incidence of postoperative retinal detachment and bacterial endophthalmitis in the Swedish national paediatric cataract register and associated risk factors

**DOI:** 10.1111/aos.17460

**Published:** 2025-02-14

**Authors:** Arzu Seyhan Karatepe Hashas, Anna Linnarsson Wiklund, Jenny Gyllén, Birgitte Haargaard, Gunilla Magnusson, Alf Nyström, Kristina Tornqvist, Eric Trocmé, Ulrika Kjellström

**Affiliations:** ^1^ Department of Ophthalmology Region Västra Götaland, Sahlgrenska University Hospital Mölndal Sweden; ^2^ Department of Clinical Neuroscience, Institute of Neuroscience and Physiology, Sahlgrenska Academy University of Gothenburg Gothenburg Sweden; ^3^ Division of Ophthalmology and Vision St Erik Eye Hospital Stockholm Sweden; ^4^ Department of Opthalmology Næstved Hospital Næstved Denmark; ^5^ Danish Serum Institute Copenhagen Denmark; ^6^ Department of Clinical Sciences, Ophthalmology, Skane University Hospital Lund University Lund Sweden

**Keywords:** bacterial endophthalmitis, congenital cataract, infantile cataract, paediatric cataract surgery, persistent fetal vasculature, postoperative complications, retinal detachment, secondary glaucoma

## Abstract

**Purpose:**

To investigate the incidence and risk factors of retinal detachment (RD) and bacterial endophthalmitis in a cohort of children who underwent cataract surgery before the age of eight.

**Methods:**

Data was retrieved from the Swedish national paediatric cataract register. All eyes with congenital or infantile cataract that underwent surgery between January 1, 2007, and December 31, 2023 with at least one follow‐up were included. Cases associated with trauma, uveitis or RD at surgery were excluded. Parameters that could be important for complications were analysed.

**Results:**

RD was found in seven of 1073 eyes reflecting an incidence of 0.65%. There were no statistically significant differences in age at surgery, presences of intellectual disability or general disease, cataract type, surgical technique, axial length, corneal diameter, previous glaucoma surgery or occurrence of persistent fetal vasculature (PFV), although the frequency of glaucoma surgery and PFV was higher in RD cases; 42.9% versus 13.2% and 57.1% versus 26.0%. Aphakia was significantly more common in RD patients; 71.4% versus 19.3% (*p* = 0.042), as well secondary glaucoma; 57.1% versus 19.5% (*p* = 0.032). No cases of endophthalmitis were observed.

**Conclusion:**

The incidence of RD was low compared to previous studies and no endophthalmitis was found. This might be a result of centralized paediatric cataract care with few but experienced surgeons. Aphakia and secondary glaucoma were associated with higher RD risk and those cases should be followed carefully. PFV and glaucoma surgery were found at a higher frequency in RD cases prompting comprehensive postoperative care also for these children.

## 1 INTRODUCTION

Childhood cataract is a treatable condition if dealt with properly with surgery in time and thorough follow‐up. Otherwise, the condition leads to significant visual impairment with considerable negative impact on both the daily life of the child and the family (Ozen Tunay et al., 2021; Teoh et al., 2021). In Sweden, the incidence of congenital cataract has been estimated to 36 cases per 100 000 live births (Abrahamsson et al., 1999) which means that approximately 40 babies a year are born with cataract of some degree and the frequency of cataract surgery in children aged 0–7 years was 31/100 000 in a previous national Swedish study (Magnusson et al., 2018). To enable an improved understanding of the different aspects of childhood cataract, for example incidence, results of surgery and follow‐up and complication rates, a national quality register, the Swedish national paediatric cataract register (PECARE), was initiated in Sweden in 2006 (Magnusson et al., 2018).

Cataract surgery is, in most cases of childhood cataract, essential for optimizing visual development, but also associated with the risk of several complications that can interfere with optimal visual outcome. The most prevalent complications are visual axis opacification (VAO) and secondary glaucoma. VAO has been described in a frequency of 5%–44% in different studies (Magnusson et al., 2024; Nyström et al., 2018; Solebo, Rahi and British Congenital Cataract Interest, 2020; Trivedi et al., 2004, 2011). The incidence of secondary glaucoma also ranges over a broad scale from 5% to 45% (Freedman et al., 2015; Murphy et al., 2020; Nyström et al., 2020; Solebo et al., 2018; Vasavada et al., 2018) and is usually encountered in higher frequencies in aphakic (12%–45%) than in pseudophakic eyes (5%–19%; Murphy et al., 2020; Solebo et al., 2018; Vasavada et al., 2018). Endophthalmitis and retinal detachment (RD) are rarer, but even more serious and sight threatening complications. The incidence of endophthalmitis has been reported to be 0.045%–0.5% (Agarkar et al., 2016; Good et al., 1990; Nguyen et al., 2021; Wheeler et al., 1992) after paediatric cataract surgery. Yet other surveys have, fortunately, reported no cases of endophthalmitis. (Boonstra & Haugen, 2022; Bothun et al., 2020; Plager et al., 2020). Concerning RD, incidence figures range from 0.8% to 7.0% (Agarkar et al., 2018; Chrousos et al., 1984; Haargaard et al., 2014; Oke et al., 2022; Plager et al., 2020; Rabiah et al., 2005). In some of the studies, parameters associated with an increased risk of RD were identified, and among these, the most frequent were persistent fetal vasculature (PFV; Chrousos et al., 1984; Oke et al., 2022; Plager et al., 2002), other ocular abnormalities (Haargaard et al., 2014), intellectual disability (Agarkar et al., 2018; Chrousos et al., 1984; Haargaard et al., 2014), myopia (Chrousos et al., 1984), prematurity (also without retinopathy; Oke et al., 2022) and aphakia (Oke et al., 2022).

The aim of this study was to investigate the cumulative incidence and timing of RD and endophthalmitis as well as the associated risk factors for these complications in a Swedish cohort of children operated for paediatric cataract before 8 years of age based on data from PECARE.

## 2 METHODS

PECARE is a subdivision of the Swedish National Cataract Register, a web‐based national surgical registry collecting data on all cataract surgeries in Sweden with the purpose to evaluate and improve cataract surgery on a national basis and to enable improved equality of cataract care in Sweden. In the paediatric sub‐register, information concerning cataract surgery in children up to 8 years of age (0–7 years) at surgery is compiled. A broad range of data describing demographics, pre‐surgical parameters (e.g. various eye measurements, symptoms and signs leading to referral, heredity etc.) and surgical parameters are collected in the register. The register also gathers data on follow‐up results from examinations at 1, 2, 5 and 10 years of age continuously documenting complications and visual outcome for each child. This setup means that at a certain timepoint, results from all follow‐ups can be available only for the children that had surgery at a young age and many years ago. For the ones that had surgery more recently or at an older age only the results for the past follow‐up timepoints can yet have been registered. PECARE started in the autumn of 2006 (Magnusson et al., 2018) and since 2007 data reflecting complete age groups are available. Over the years, the registration frequency in PECARE has been high, ranging between 85% and 100% from 2015 and onwards (https://rcsyd.se/pecare). In Sweden, surgical intervention for cataract in children under the age of three are centralized to two national highly specialized care units in Gothenburg and Stockholm. These units employ a select group of surgeons who have received specialized training in paediatric cataract surgery. This organization of care has been applied since 2012. In practice, almost all children between three and 8 years of age are also referred to these two national highly specialized care units when in need of cataract surgery.

In this study, PECARE data on paediatric cataract surgeries performed in Sweden, from January 1, 2007, to December 31, 2023, was analysed retrospectively to investigate the incidence of postoperative RD and bacterial endophthalmitis. Data extraction was made from the registry in February 2024. Eyes with cataract associated with trauma or uveitis and eyes with RD already at the primary cataract surgery were excluded from the study. Moreover, it was mandatory for inclusion to have attended at least one of the follow‐up visits recorded in the register at 1, 2, 5 and 10 years of age (a manual for the register is available at https://rcsyd.se/pecare/vardpersonal/formular‐och‐manualer/attachment/manual‐barnkataraktregistret‐2024). Registered complications had developed during the period since the previous registration; for example, a certain complication such as secondary glaucoma that were registered at 2 years of age had not been detected at 1 year of age but had developed since the previous registration. The follow‐up frequency is shown in Table 1. The percentages reflect a snapshot of the PECARE register in February 2024 when the data were extracted. Eyes with RD identified during follow‐up after the primary surgery are included in the ‘Case’ group, and the remaining eyes are in the ‘Control’ group. Demographic data such as age at surgery, gender, uni‐ or bilateral disease and heredity were analysed. Moreover, factors that might be associated with an increased risk of RD and endophthalmitis were studied. These were the type of cataract, axial length, corneal diameter, associated general disorders, the presence of intellectual disability, data concerning surgical technique, aphakia/pseudophakia, the presence of secondary glaucoma, past glaucoma surgery and concurrent ocular abnormalities such as PFV, microcornea and microphthalmos.

**TABLE 1:** Data on frequency of follow‐up registrations at 1, 2, 5 and 10 years of age in the control group.

Number of eyes with surgery <1 year of age	527
Follow‐up at 1 year of age	527 (100.0%)
Surgery <1 year of age but no 1‐year follow‐up data in the register	0 (0.0%)
Number of eyes with surgery <2 years of age	608
Follow‐up at 2 years of age	585 (96.2%)
Surgery <2 years of age but no 2‐years follow‐up data in the register	23 (3.8%)
Number of eyes with surgery <5 years of age	765
Follow‐up at 5 years of age	733 (95.8%)
Surgery <5 years of age but no 5‐years follow‐up data in the register	32 (4.2%)
Number of eyes with surgery <8 years of age	575
Follow‐up at 10 years of age	507 (88.2%)
Surgery <8 years of age but no 10‐years follow‐up data in the register	68 (11.8%)

*Note*: For each follow‐up timepoint (1, 2, 5 and 10 years), only the eyes of children that have had surgery before the given age and that also have reached the given follow‐up age are included. Thus, fewer eyes are available for the follow‐up at 10 years of age.

The study was conducted in accordance with the tenets of the Declaration of Helsinki and was approved by the Ethics Review Authority in Sweden (ref: 2023‐07821‐02).

### 2.1 Statistical analysis

Continuous variables were summarized with, n (number of observations), mean, standard deviation (SD), 95% confidence interval, median and range (minimum and maximum). Additionally, a two‐sided non‐parametric test, the Wilcoxon rank sum test, was used to test the null hypotheses that the two groups are similar, against the alternative hypothesis that there is a difference between the groups.

Categorical variables were summarized with *n* (number of observations), and percentage. Additionally, a two‐sided Fisher's exact test, was used to test the null hypotheses that the two groups are similar with regards to the distribution of responses, against the alternative hypothesis that there is a difference between the groups.

A *p*‐value below 0.05 was considered statistically significant. However, due to the limited number of observations, caution should be exercised when drawing conclusions from the study.

The frequency of RD over time after cataract surgery was illustrated with Kaplan–Meier plots.

SAS® Viya® 3.5 was used for the statistical computations.

## 3 RESULTS

**FIGURE 1:**
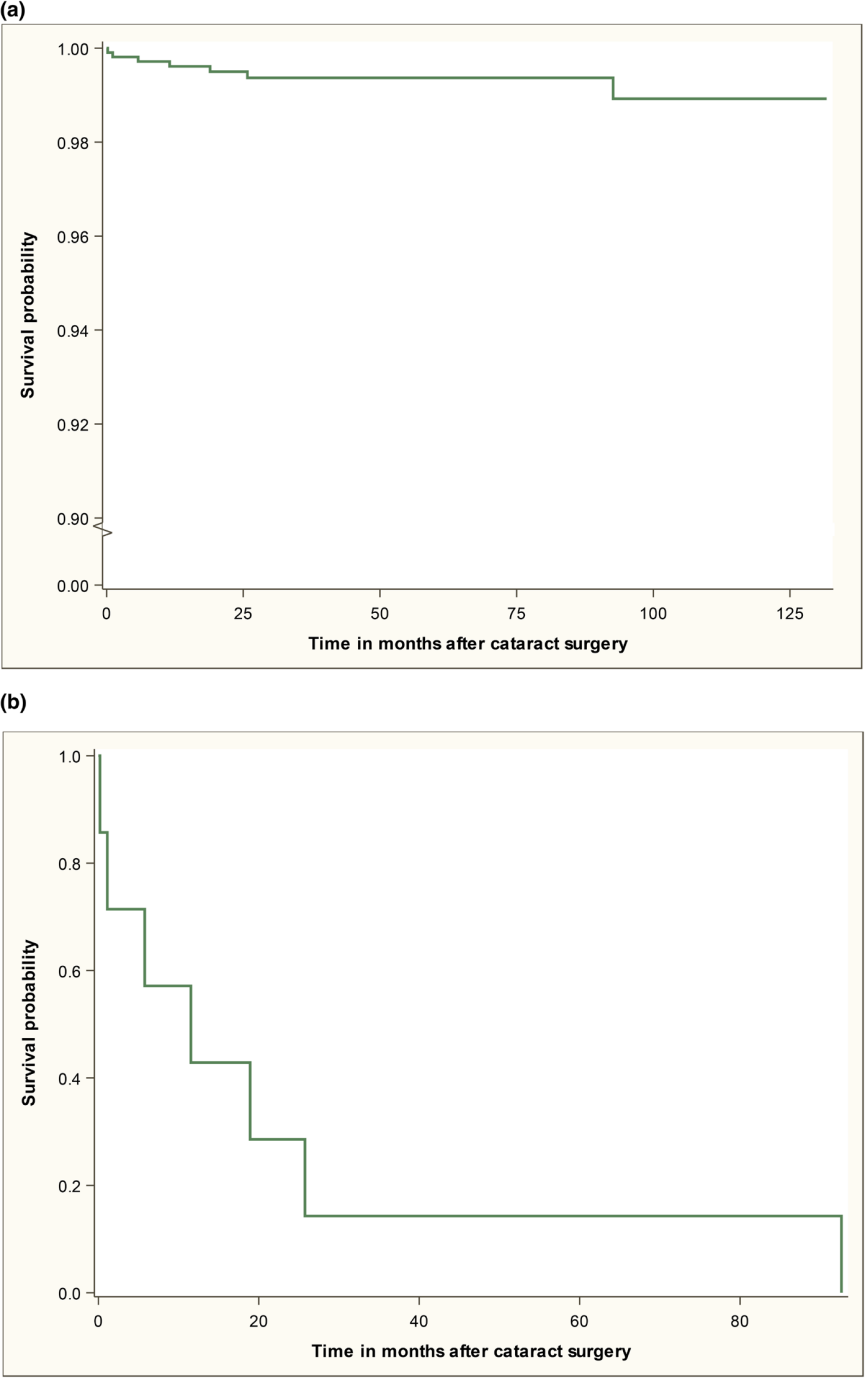
(a) Illustrating the frequency of retinal detachment (RD) in the patient material with a Kaplan–Meier plot. Since the RD cases are very few compared to the total number of patients, the slope of the curve, reflecting cases who have fallen ill with RD, is very flat. In (b) only the RD cases are included demonstrating that most RDs are encountered during the first 2 years after cataract surgery.

By December 31, 2023, a total of 1345 eyes belonging to 970 children, 459 girls (47.3%) and 511 boys (52.7%) were registered in PECARE. Of these, 227 eyes had not yet been followed up, 23 eyes were excluded due to traumatic cataract, 18 eyes were excluded since the cataract was associated with uveitis and four eyes were excluded because RD was encountered at the primary cataract surgery. This leaves 1073 eyes in 752 children, 351 (46.7%) girls and 401 (53.3%) boys to be included in our study. RD was found in seven of the 1073 eyes during the follow‐up period reflecting an incidence of 0.65%. The frequency of RD in the patient material (including both controls and RD cases), is illustrated with a Kaplan–Meier plot in Figure 1a. No verified cases of bacterial endophthalmitis were identified. The RD cases had been followed for a mean time of 47.0 ± 43.6 (SD) months (95% confidence interval (CI) 6.6–87.3), median 23 (range 9–120) months. The corresponding mean follow‐up time for controls was 56.7 ± 37.3 months (95% CI 54.4–58.9) with median 54 (0–134). There was no statistically significant difference between the groups, *p* = 0.341.

Patients with RD underwent cataract surgery at the mean age of 19.1 ± 29.9 months (95% CI ‐8.5‐46.8) and median age was one month (0–73) compared to mean age 25.2 ± 28 months (95% CI 23.5–26.9) with median 13 (0–100) months in the control cohort. There was no statistically significant difference in median age at surgery between the groups, *p* = 0.337 (see Table 2 summarizing data on age in months and weeks). Table 3 shows age distribution for RD cases and control eyes divided into three age groups; <3, 3–12 and >12 months, revealing that most RD cases (57.1%) underwent cataract surgery when they were younger than 3 months of age. The corresponding percentage for control eyes was 37.2%.

**TABLE 2:** Statistics on age, axial length and corneal diameter at the time of cataract surgery in cases with retinal detachment (RD) versus controls.

Variable	Statistics	RD cases	Controls
Age in months	*n*	7	1066
	Mean (SD)	19.1 (29.9)	25.2 (28.0)
	95% CI	(−8.5–46.8)	(23.5–26.9)
	Median	1	13
	(min–max)	(0–73)	(0–100)
	*p*‐Value		0.3370
Age in weeks	*n*	7	1066
	Mean (SD)	85.0 (129.4)	111.4 (121.9)
	95% CI	(−34.7–204.7)	(104.1–118.7)
	Median	7	57
	(min–max)	(2–317)	(1–438)
	*p*‐Value		0.3098
Axial length (mm)	*n*	7	1032
	Mean (SD)	19.46 (3.82)	19.53 (2.75)
	95% CI	(15.93–22.99)	(19.36–19.69)
	Median	21.37	19.89
	(min–max)	(13.73–23.68)	(11.40–28.30)
	*p*‐Value		0.8083
Corneal diameter (mm)	*n*	6	942
	Mean (SD)	10.02 (1.79)	10.70 (1.23)
	95% CI	(8.14–11.90)	(10.62–10.78)
	Median	9.7	11.0
	(min–max)	(8.3–12.0)	(6.0–16.1)
	*p*‐Value		0.3632

**TABLE 3:** Age distribution in eyes with retinal detachment (RD) versus control eyes.

Age	RD cases		Controls		Total	
	*n*	%	*n*	%	*n*	%
<3 months	4	57.1	397	37.2	401	37.4
3–12 months	1	14.3	130	12.2	131	12.2
>12 months	2	28.6	539	50.6	541	50.4
Total	7	100.0	1066	100.0	1073	100.0

*Note*: Age groups according to the following: 0–90 days: <3 months; 91–365 days: 3–12 months; >365 days: >12 months.

Abbreviation: *n*, number of eyes.

The mean axial length in the RD‐group was 19.46 ± 3.82 mm (95% CI 15.93–22.99), median 21.37 mm (13.73–23.69) and the mean horizontal corneal diameter was 10.02 ± 1.79 mm (95% CI 8.14–11.90), median 9.7 mm (8.3–12.0). In the control cohort, corresponding values were mean 19.53 ± 2.75 mm (95% CI 19.36–19.69), median 19.89 mm (11.40–28.30) for the axial length and mean 10.70 ± 1.23 mm (95% CI 10.62–10.78), median 11.0 mm (6.0–16.1) for the corneal diameter. There were no statistically significant differences between the groups concerning axial length or horizontal corneal diameter (see Table 2 summarizing data concerning age, axial length and corneal diameter).

**FIGURE 2:**
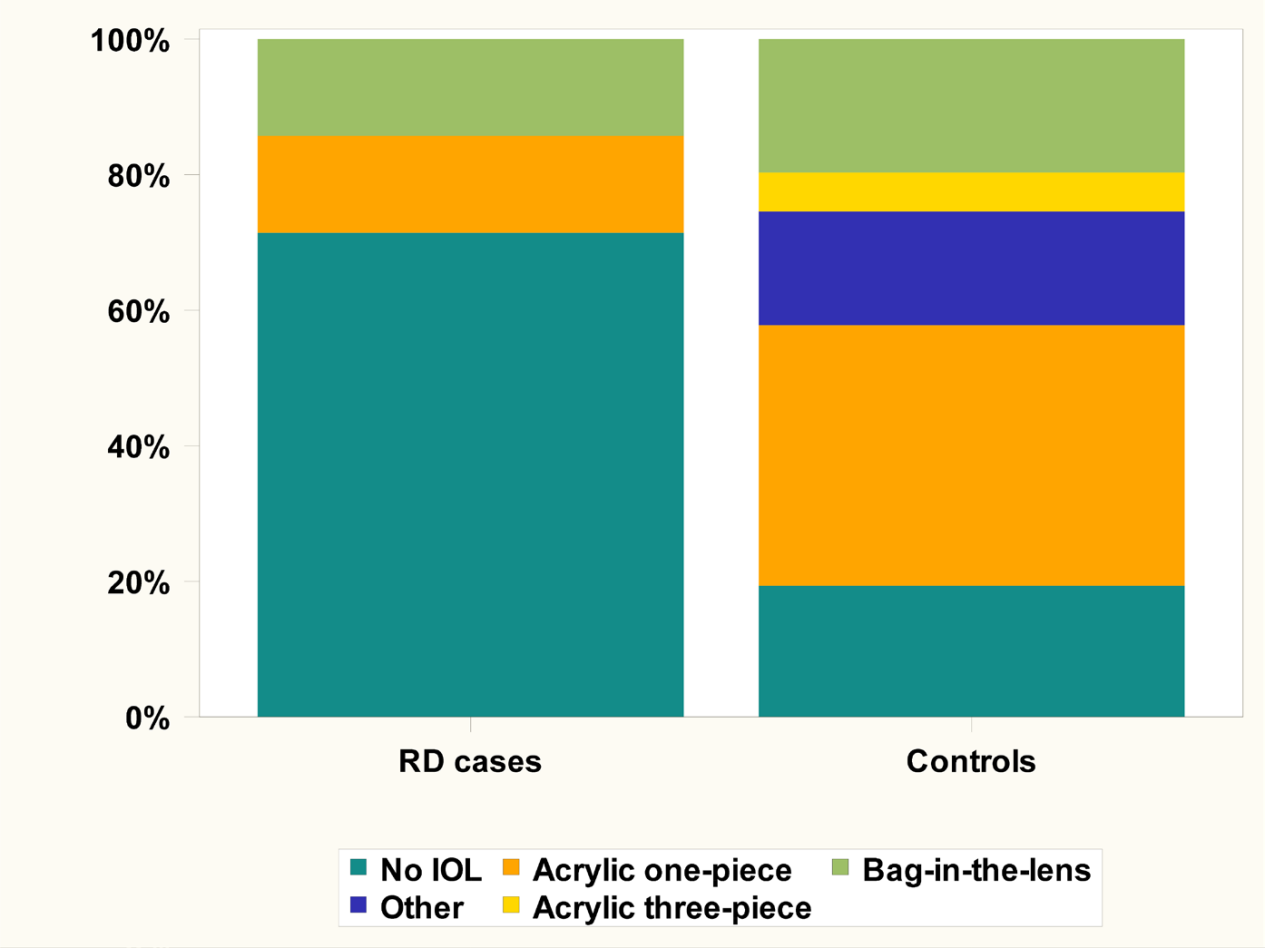
Showing what kind of intraocular lenses (IOL) that were implanted at the primary surgery. In cases with retinal detachment (RD), the majority of eyes were left aphakic.

Among the RD cases, four out of seven (57.1%) had PFV of some degree and two (28.6%) had developed secondary glaucoma that needed surgical treatment before the diagnosis of RD was made. Moreover, one of the RD eyes (in case 5) was affected by congenital glaucoma that had been treated surgically before cataract surgery and before the RD was diagnosed (see description of cases below and in Table 4). Thus, all in all three out of seven (42.9%) RD cases had had glaucoma surgery. Over the entire follow‐up period, four of the RD eyes (57.1%) developed secondary glaucoma that needed treatment of any kind, medical and/or surgical (Table 4). In the control cohort, 277 eyes (26.0%) had PFV, 141 eyes (13.2%) had had glaucoma surgery, and 208 eyes (19.5%) had secondary glaucoma of any kind (that needed medical and/or surgical treatment). Statistical comparison showed no significant differences concerning PFV prevalence (*p* = 0.130) or frequency of glaucoma surgery (*p* = 0.055) (although borderline), while secondary glaucoma was more frequent in RD eyes than in the control cohort (*p* = 0.032). Five of the RD cases (71.4%) were left aphakic at the primary surgery and two of them (28.6%) received an IOL (one got a bag‐in‐the lens IOL (BIL‐IOL) and the other a one‐piece acrylic IOL). Moreover, five of the RD patients (71.4%) presented with unilateral cataract while two of them (28.8%) had bilateral disease. In the control cohort, 206 (19.3%) eyes were left aphakic and 860 (80.7%) received an IOL at the primary surgery. This means that a statistically significant higher proportion of patients among the RD cases than controls were left aphakic (*p* = 0.004). 366 control cases (34.3%) were unilateral and 700 (65.7%) were bilateral and this did not differ between the groups. Concerning density ‐ and type of cataract, presence of other eye malformations and surgical technique, there were no differences between the groups. Data on the cataract itself, and concomitant ophthalmological morbidity are presented in Table 5. Figure 2 visualizes the distribution of different lenses or aphakia among RD cases and controls.

**TABLE 4:** Summarizing demographic and ophthalmic data concerning the cases suffering from retinal detachment (RD).

Patient	Sex (F/M)	Other disease	Age at cataract detection	Eye (R/L/B)	Axial length (mm)	Corneal diameter (mm)	Age at cataract surgery	IOL	Ocular malformation	RD (R/L)	Age at RD	Secondary glaucoma	Glaucoma surgery before RD
1	M	Gorlin syndrome	At birth	R	21.37	10.8	7 weeks	No	PFV	R	12 months	Yes, diagnosed also after RD, treated pharmacologically	No
2	M	No	At birth	R	13.73	8.5	3 weeks	No	PFV, microphthalmia	R	5 weeks	No	No
3	F	No	At birth	B	16.75/16.29	8.5/9.0	3 weeks	No	No	R	94 months	Yes	Yes
4	F	No	At birth	B	17.34/17.23	8.5/8.3	5 weeks	No	No	L	20 months	Yes	Yes
5	M	No	10 months	R	23.68	–	41 weeks	No	Lens malformation	R	16 months	No	Yes, but for congenital glaucoma
6	M	No	35 months	R	22.37	12.0	51 months	Yes	PFV	R	52 months	Yes, treated pharmacologically	No
7	M	No	72 months	L	22.10	12.0	73 months	Yes	PFV, iris atrophy	L	80 months	No	No

Abbreviations: B, both eyes; F, female; IOL, intraocular lens; L, left eye; M, male; mm, millimetre; PFV, persistent fetal vasculature; R, right eye.

**TABLE 5:** Statistics on variables associated with the cataract itself and concomitant ophthalmological morbidity in retinal detachment (RD) cases versus controls.

Variable	Categories	RD cases	Controls
Laterality of the cataract: *n* (%)	Unilateral	5 (71.4)	366 (34.3)
	Bilateral	2 (28.6)	700 (65.7)
*p*‐Value			0.0535
Intraocular lens: *n* (%)	No	5 (71.4)	206 (19.3)
	Yes	2 (28.6)	860 (80.7)
*p*‐Value			*0.0042*
Dense cataract: *n* (%)	Yes	4 (57.1)	670 (62.9)
	No	3 (42.9)	396 (37.1)
*p*‐Value			0.7154
Persistent fetal vasculature: *n* (%)	Yes	4 (57.1)	277 (26.0)
	No	3 (42.9)	781 (73.3)
	Unknown	0 (0.0)	8 (0.8)
*p*‐Value			0.1303
Secondary glaucoma any time during follow‐up: *n* (%)	Yes	4 (57.1)	208 (19.5)
	No	3 (42.9)	858 (80.5)
*p*‐Value			*0.0315*
Surgery for glaucoma: *n* (%)	Yes	3 (42.9)	141 (13.2)
	No	4 (57.1)	925 (86.8)
*p*‐Value			0.0550
Other eye malformations: *n* (%)	Yes	3 (42.9)	223 (20.9)
	No	4 (57.1)	843 (79.1)
*p*‐Value			0.1662

Statistically significant *p*‐values are given in italics.

Only one of the seven cases with RD (14.3%) suffered from a general disease (Gorlin syndrome) also leading to intellectual disabilities, while the other six cases had an isolated eye disorder (Table 6). In the control cohort, 251 (23.5%) cataract cases were associated with general disease and 146 (13.7%) with intellectual disability. Data concerning intellectual disability was missing in 46 (4.3%) of the control cases. The was no statistically significant difference in the prevalence of general disease or intellectual disability between RD patients and controls (*p* = 0.210 and *p* = 1.000, respectively).

**TABLE 6:** Statistics on other eye diseases and intellectual disability during follow‐up in cases with retinal detachment (RD) versus controls.

Variable	Categories	RD cases	Controls
Other eye diseases any time at follow‐up: *n* (%)	Missing	0 (0.0)	48 (4.5)
	Yes	7 (100.0)	195 (18.3)
	No	0 (0.0)	817 (76.6)
	Unknown	0 (0.0)	6 (0.6)
*p*‐Value			*<0.0001*
Intellectual disability: *n* (%)	Missing	0 (0.0)	46 (4.3)
	Yes	1 (14.3)	146 (13.7)
	No	6 (85.7)	825 (77.4)
	Unknown	0 (0.0)	49 (4.6)
*p*‐Value			1.0000

In the RD patients, the diagnosis of retinal detachment was made within a mean time of 22.3 ± 32.4 months (95% CI −7.7 to 52.2), median 11.5 (0.2–92.6) from the primary cataract surgery. This is demonstrated with a Kaplan–Meier plot in Figure 1b.

### 3.1 Description of RD cases (summarized in Table 4)

*Case 1* is a boy, born at term who was diagnosed with Gorlin syndrome, a condition caused by a pathogen heterozygous variant in the *PTCH1* gene. In addition to the cataract, he was born with syndactyly, retentio testis, benign enlargement of subarachnoid space in infancy (BESS), attenuated corpus callosum, small pituitary gland and macrocephaly and thus has an intellectual disability. At 7 weeks of age, his right eye was operated for cataract with central clouding of the lens and a quite extensive PFV that was adherent both to the optic nerve and the retina. 52 weeks after cataract surgery a change of iris colour was noted, and the consequent examination revealed a RD. During his second year of life, the boy developed secondary glaucoma that was treated pharmacologically. Visual outcome was poor ending up with amaurosis.

*Case 2* is a boy, born at term with low‐grade clouding of the lens in his right eye, but a dense vascularized retrolental membrane and microphthalmos. Cataract extraction was performed at 3 weeks of age and RD was diagnosed 6 days after primary surgery. It was noticed due to the relapse of a dense retro pupillary membrane. The visual outcome was poor with amaurosis.

*Case 3* is a girl born at term with bilateral nuclear cataracts. Bilateral cataract surgery was carried out at 3 weeks of age. During her first year of life, the right eye developed secondary glaucoma which had to be treated extensively both pharmacologically and surgically. Diode laser treatment was performed six times between the age of 4–54 months, a trabeculotomy was carried out at 20 months of age and she received a Molteno tube implant when she was 24 months old. At the age of 93 months the Molteno tube implant required revision surgery. Nine days later, she came back with nausea and vomiting, and examination revealed high intraocular pressure, subretinal bleeding and RD. Despite several retinal surgeries, the visual outcome in the right eye was poor with VA 0.05 (decimal letter chart).

*Case 4* is a girl, born at term with bilateral nuclear cataracts. She underwent cataract surgery at 5 weeks of age. During her first year of life, secondary glaucoma was diagnosed in the left eye which had to be treated both pharmacologically and surgically. A trabeculotomy was carried out when she was 17 months old. The surgery was complicated by a vitreous bleeding which was diagnosed on the first postoperative day. Thirteen weeks later, the red reflex was still blurred. She was, therefore, re‐examined under general anaesthesia and a RD was detected (19 months after cataract extraction). The visual outcome of the left eye was eventually poor with VA 0.02 on a decimal chart.

*Case 5* is a boy, born at term. At 29 weeks of age, infantile glaucoma and infantile cataract with lens malformation, and defective zonulas were diagnosed in his right eye. Immediately at diagnosis, trabeculotomy was carried out and after surgery, he was prescribed dorzolamide, timolol and latanoprost. Cataract surgery was performed at 41 weeks of age. At 12 months of age, he received an Ahmed shunt. A little less than 4 months later, (almost 6 months after the primary cataract surgery), RD was diagnosed on a routine follow‐up. Visual outcome of the right eye was poor with amaurosis.

*Case 6* is a boy, born at term. At 35 months of age, he was referred to an ophthalmological department due to a squint. The examination revealed right eye juvenile cataract combined with PFV including a dense vessel and a fibrotic stalk connecting the optic nerve head and the cataractous lens. The dense PFV membrane also tended to lift the retina around the optic nerve head. He underwent cataract surgery at 51 months of age. Postoperative treatment included brinzolamide drops to reduce the intraocular pressure. 14 days after cataract surgery he complained of reduced vision and RD was detected. The visual outcome was poor with VA 0.07 on a decimal letter chart.

*Case 7* is a boy, born at term who was 73 months old when he underwent surgery for juvenile cataract in his left eye. He was referred to the ophthalmology department due to grey discoloration of the pupil and was found to have a dense nuclear cataract, PFV with persistent arteria hyaloidea and iris atrophy. 26 weeks after the cataract surgery RD was diagnosed at a regular follow‐up visit. Visual outcome was poor with VA 0.01 on a decimal chart.

## 4 DISCUSSION

Childhood cataract is a sight threatening but treatable condition if dealt with in time and with proper actions; often including surgery. To improve medical care, it is important to evaluate the risks of different postoperative complications that can impair the prerequisites for optimal visual outcome and also, to update clinical procedures based on the new knowledge to minimize complications. In congenital cataract, it is well known that VAO and secondary glaucoma can disturb postoperative status in 5%–44% (Magnusson et al., 2024; Nyström et al., 2018; Solebo, Rahi and British Congenital Cataract Interest, 2020; Trivedi et al., 2004, 2011) and 5%–45% (Freedman et al., 2015; Murphy et al., 2020; Nyström et al., 2020; Solebo et al., 2018; Vasavada et al., 2018) of the eyes, respectively, and previous studies have also indicated that the use of BIL‐IOLs (Boonstra & Haugen, 2022; Magnusson et al., 2024; Nyström et al., 2018) or leaving the eyes aphakic (Bothun et al., 2020; Solebo et al., 2018) may reduce the risk of VAO, thus helping in improving the cataract care. Less is known about postoperative RD and bacterial endophthalmitis after paediatric cataract surgery. Therefore, the aim of this study was to investigate the incidence of these complications and risk factors associated with them in the Swedish national paediatric cataract register, PECARE, that include children operated for cataract before the age of eight.

No cases of culture positive bacterial endophthalmitis were identified in our cohort and this is in line with some previous studies (Boonstra & Haugen, 2022; Bothun et al., 2020; Plager et al., 2020; Solebo et al., 2018). In other investigations though, the incidence of endophthalmitis has been reported to be 0.045%–0.5% (Agarkar et al., 2016; Good et al., 1990; Nguyen et al., 2021; Wheeler et al., 1992), which is slightly higher than in adults with an reported incidence of 0.035%–0.050% (Lundstrom et al., 2015; Shao et al., 2021) highlighting the importance of keeping to strict hygiene guidelines, including the routine use of intracameral antibiotics (Wejde et al., 2005) in order to minimize the risk of endophthalmitis.

Moreover, the incidence of RD was 0.65% in our cohort correlating well with the incidence 0.8% found by Haargaard et al., 2014 after an observation time of five years. Yet other studies (Agarkar et al., 2018; Chrousos et al., 1984; Oke et al., 2022; Plager et al., 2020; Rabiah et al., 2005) have presented higher incidence rates ranging between 1.5% and 5.5%. A difference between these studies and ours is that they also included older children and teenagers up to 15–19 years, while our patients were under 8 years of age at surgery. On the other hand, age at surgery has not been statistically correlated with higher risk of RD, neither in previous studies (Agarkar et al., 2018; Haargaard et al., 2014; Oke et al., 2022) nor in our material even though a higher proportion, 71.4%, of our RD cases were operated during their first 12 months of life than at older ages, 28.6% and corresponding distribution among the controls were 48.2% versus 51.8% (see Table 3). Another aspect that might explain the differences in incidence is follow‐up time. Our patients had been followed for a mean time of 4.65 years when data was evaluated. Most of the other studies had a similar follow‐up times of 5–5.5 years (Agarkar et al., 2018; Chrousos et al., 1984; Haargaard et al., 2014; Oke et al., 2022), while three of them had followed their patients longer; from 6.8 to 20 years (Haargaard et al., 2014; Plager et al., 2020; Rabiah et al., 2005) and of course thereby have a higher chance of picking up later cases of RD with these extended observation times. Looking into our material though, six out of seven (86%) RD cases, as shown by Figure 1b, were diagnosed with the RD within the first 2 years after cataract surgery and thus hopefully we have caught the absolute majority of RD cases despite the follow‐up time. Regarding the quite low RD incidence of 0.65% in our study, the organization of childhood cataract surgery in Sweden with two centralized care units with few but very experienced surgeons performing the surgical procedures, might be beneficial for avoiding RD after paediatric cataract surgery.

In the study, we also intended to evaluate pre‐, intra‐ and postoperative factors that might be associated with an increased risk of RD. Concerning specific ocular characteristics (Table 5), neither type of cataract (lamellar, nuclear, posterior subcapsular, anterior subcapsular) or the presence of dense cataract, nor axial length, corneal diameter (as an indicator of microphthalmos) (Table 2) or presence of PFV (Table 5) were significantly associated with RD occurrence, although PFV was found in higher frequency in RD patients (51.7%) than in controls (26.0%). Increased rate of PFV has been described as a risk factor for RD in childhood cataract surgery also by other authors (Chrousos et al., 1984; Oke et al., 2022; Plager et al., 2002). When it comes to axial length and corneal diameter extra caution should be made in the interpretation of these results since a greater share of the RD cases were operated during their first 3 months of life (57.1%) compared to the controls (36.2%) (Table 3) even though there was no statistically significant difference in age at surgery (Table 2). Generally, axial length and corneal diameter are expected to be smaller in younger children than in older ones and if the dimensions do not differ, this could indicate that they are larger than normal in the younger group, although not statistically proven. Thus, a separate study including age‐matched controls is of future interest for this certain issue. Indeed, longer axial length has been associated with increased RD risk in a previous study by Agarkar et al. (2018).

We found no differences in the frequency of anterior vitrectomy between cases with and without RD, but a statistically significant larger proportion of the patients with RD than control cases were left aphakic, 71.4% versus 19.3% (Table 5). Aphakia has been correlated to increased RD risk also in another study (Oke et al., 2022) and it might be a combination of the aphakia itself (leading to more mobility of the vitreous) and the fact that more complicated cases usually are the ones left aphakic that contribute to the increased risk. This may be supported by the fact that other eye diseases of any kind during the follow‐up period were significantly more common in RD cases than in the control cohort (Table 6). It is an interesting question whether it is the aphakia itself that is a risk factor for RD or if the risk factors include the conditions that require leaving the eye aphakic during surgery. The most important reason for leaving the eye aphakic during paediatric cataract surgery is that the eye is too small to implant an intraocular lens. However, the fact that there was no statistical difference in corneal diameter or axial length between the eyes that developed RD and the control group in our analysis leads us to believe that aphakia itself is an independent risk factor for RD.

Another parameter significantly associated with increased risk of RD was secondary glaucoma that was found in 51.7% of RD cases while only 19.5% of control cases were affected. Three of the seven RD patients (cases 3, 4 and 5) had had glaucoma surgery (cases 3 and 4 for secondary glaucoma and case 5 for congenital glaucoma). In all these three patients, the RD was diagnosed after both the cataract ‐ and glaucoma surgeries had been carried out. Hence, the glaucoma surgery could as well be a contributing cause of RD in these patients although the statistical comparison of the frequency of surgeries did not quite reach significance (Table 3). Nevertheless, previous glaucoma surgery was more common in RD cases than in controls; 42.9% versus 13.2%.

In some former surveys, intellectual disability (Agarkar et al., 2018; Chrousos et al., 1984; Haargaard et al., 2014) and prematurity (Oke et al., 2022) have been overrepresented in patients with RD after cataract surgery but this was not the case in our study.

To our knowledge, this is one of the larger studies investigating the incidence of postoperative RD and bacterial endophthalmitis on a national level. Still, due to the small number of observations of RD and no endophthalmitis, caution should be exercised when drawing conclusions from the results. To compensate as much as possible for the differences in size of the two cohorts (RD cases and control cases) non‐parametric statistical tests have been used. Another drawback of the study is the wide distribution of duration of follow‐up between patients, which is due to the use of register data and to the setup of the register. Of course, longer and more similar observation times for all children, as in some of the other studies (Haargaard et al., 2014; Plager et al., 2020; Rabiah et al., 2005), would have been beneficial for picking up further cases of RD, likely leading to a higher incidence compared to our present study results, and therefore, we plan to continue the PECARE follow‐up to obtain and report additional data by time. Nevertheless, this is a large study made on a national basis and thereby we hope that it can still contribute with important data.

In conclusion the incidence of RD in PECARE is low, 0.65% and no cases of bacterial endophthalmitis were found, indicating that centralized and specialized surgical care of childhood cataract is beneficial to avoid these complications. Aphakia, secondary glaucoma and other eye diseases in combination with the cataract were associated with statistically significant increased risk of RD and those cases should be followed extra carefully. Moreover, PFV and previous glaucoma surgery were found at a higher frequency in RD cases than in the control cohort prompting careful postoperative care also of eyes with these conditions.
